# Evaluation of Mid-Infrared and X-ray Fluorescence Data Fusion Approaches for Prediction of Soil Properties at the Field Scale

**DOI:** 10.3390/s23020662

**Published:** 2023-01-06

**Authors:** Isabel Greenberg, Michael Vohland, Michael Seidel, Christopher Hutengs, Rachel Bezard, Bernard Ludwig

**Affiliations:** 1 Department of Environmental Chemistry, University of Kassel, 37213 Witzenhausen, Germany; 2 Geoinformatics and Remote Sensing, Institute for Geography, Leipzig University, 04103 Leipzig, Germany; 3 Remote Sensing Centre for Earth System Research, Leipzig University, 04103 Leipzig, Germany; 4 German Centre for Integrative Biodiversity Research (iDiv) Halle-Jena-Leipzig, 04103 Leipzig, Germany; 5 Department of Geochemistry and Isotope Geology, University of Göttingen, Goldschmidtstrasse 1, 37077 Göttingen, Germany

**Keywords:** cation exchange capacity, labile carbon, MIR, model averaging, multi-sensor fusion, pH, proximal soil sensing, spectroscopy, texture, XRF

## Abstract

Previous studies investigating multi-sensor fusion for the collection of soil information have shown variable improvements, and the underlying prediction mechanisms are not sufficiently understood for spectrally-active and -inactive properties. Our objective was to study prediction mechanisms and benefits of model fusion by measuring mid-infrared (MIR) and X-ray fluorescence (XRF) spectra, texture, total and labile organic carbon (OC) and nitrogen (N) content, pH, and cation exchange capacity (CEC) for *n* = 117 soils from an arable field in Germany. Partial least squares regression models underwent a three-fold training/testing procedure using MIR spectra or elemental concentrations derived from XRF spectra. Additionally, two sequential hybrid and two high-level fusion approaches were tested. For the studied field, MIR was superior for organic properties (ratio of prediction to interquartile distance of validation (RPIQV) for total OC = 7.7 and N = 5.0)), while XRF was superior for inorganic properties (RPIQV for clay = 3.4, silt = 3.0, and sand = 1.8). Even the optimal fusion approach brought little to no accuracy improvement for these properties. The high XRF accuracy for clay and silt is explained by the large number of elements with variable importance in the projection scores >1 (Fe ≈ Ni > Si ≈ Al ≈ Mg > Mn ≈ K ≈ Pb (clay only) ≈ Cr) with strong spearman correlations (±0.57 < r_s_ < ±0.90) with clay and silt. For spectrally-inactive properties relying on indirect prediction mechanisms, the relative improvements from the optimal fusion approach compared to the best single spectrometer were marginal for pH (3.2% increase in RPIQV versus MIR alone) but more pronounced for labile OC (9.3% versus MIR) and CEC (12% versus XRF). Dominance of a suboptimal spectrometer in a fusion approach worsened performance compared to the best single spectrometer. Granger-Ramanathan averaging, which weights predictions according to accuracy in training, is therefore recommended as a robust approach to capturing the potential benefits of multiple sensors.

## 1. Introduction

Soil is the foundation for global food production and also provides a number of critical environmental services, such as regulating the hydrological cycle, serving as habitat, and mitigating climate change through C sequestration [1]. However, due to the heterogeneous and dynamic nature of soils [2], a high spatial and temporal density of soil information is required in many contexts, including precision agriculture, soil mapping, contamination monitoring, and documentation of soil C sequestration. In this context, sensors utilizing various segments of the electromagnetic spectrum offer a rapid, non-destructive and cheaper alternative to traditional laboratory methods [3]. Following calibration with paired reference data and spectral measurements, models can be used to predict a range of soil properties. However, the resulting model accuracy depends on the prediction mechanisms for the soil property of interest [4].

The use of mid-infrared (MIR) spectroscopy (MIRS) is well-established in the field of soil science. MIRS utilizes radiation in the range of 2500–25,000 nm (4000–400 cm^−1^), which contains fundamental vibrations of many organic molecules containing soil organic carbon (OC) and nitrogen (N) as well as minerals in the clay (e.g., kaolinite, smectite) and sand (e.g., quartz) particle size fractions [5]. The proportionality and specificity of spectrally-active molecules in relation to the soil property of interest are the basis for quantitative spectral models [6], and thus affect the model accuracy that can be attained. For example, reviews have found generally higher estimation accuracy for OC, total N, clay and sand content compared to lower and more inconsistent estimation accuracy for complex properties related to both organic and inorganic soil fractions, like pH and CEC [3,7] [3,7]. Other infrared studies [8,9,10] describe such complex properties as secondary soil properties indirectly estimated by covariations with primary soil properties (e.g., OC and clay). Fraction OC contents of various residence times may also be considered indirectly estimated properties, as prediction mechanisms utilize not only unique spectral signatures of e.g., aliphatic peaks [11], but also covariations with clay minerals [12]. Given the often insufficient model accuracy and robustness for these indirectly estimated soil properties, incorporation of other predictors in addition to MIRS should be investigated. 

In this context, X-ray fluorescence (XRF) spectrometry may be of interest due to the contrasting information it provides. XRF utilizes X-rays ranging in energy from 0.1–100 keV (0.01–25 nm) [13]. These X-rays are partially absorbed by the sample material, resulting in the expulsion of electrons from the atom, followed by electrons falling from outer shells (higher energy state) to inner shells (lower energy state), with the surplus energy resulting in the emission of fluorescent X-ray radiation at discrete energies characteristic of the elemental contents of the sample [14]. Energy dispersive XRF (EDXRF) spectrometers generally have an elemental detection range of Na to U [13]. Thus, elemental contents within this range—which contains the elements dominating the soil matrix, plant macro- and micronutrients, and heavy metal contaminants—can be quantified based on the fluorescence spectra. Monitoring of heavy metals in soils is already a well-established XRF application [15,16]. However, there is also the potential to use XRF spectra or elemental contents quantified from the XRF spectra by spectral deconvolution to estimate soil properties that co-vary with total elemental contents. For example, since XRF sensors can quantify contents of elements that compose the majority of the soil matrix, attempts have been made to quantify sand and clay contents [17,18] based on the unique assemblage of elements present in coarse particles (e.g., quartz) vs. fine particles (e.g., aluminosilicate clay minerals). Attempts have also been made to quantify soil organic matter (SOM) and OC content [19,20,21], though these fractions are dominated by elements too light to be directly quantified by XRF (e.g., H, C, O). Therefore, these predictions rely on either covariations with heavier elements or scattering peaks in the case of quantification directly from the XRF spectra. Scattering is indirectly related to the average atomic number (Z) of the sample, with a larger quantity of light elements (e.g., H, C, O) resulting in a lower average Z, and thus higher scattering [13]. Additionally, efforts have been made to estimate CEC and pH [22,23,24] As with MIR, prediction mechanisms for these properties may be increasingly complex, given the influence of both organic and inorganic soil fractions. Collectively, these studies have demonstrated the potential of expanded use of XRF in the field of soil science, with many models achieving satisfactory to excellent performance. However, studies have often focused more on model performance and less on a process-based justification for use of the method or investigation of the underlying mechanisms that enable prediction. Thus, much remains to be understood about the prediction mechanisms for XRF models quantifying soil organic and inorganic fractions, as well as other complexly related soil properties, in order to judge the reliability of such models. 

In addition to the separate use of spectrometers, there is potential to increase the scope and accuracy of soil characterization through sensor fusion due to the contrasting information collected by infrared and XRF spectrometers. Several studies have tested this hypothesis, pairing XRF with MIRS [22], visible/near-infrared spectroscopy (visNIRS) [18,25,26,27], or both [19,20]. Results regarding the benefits of model fusion have been contradictory. For example, improvements to prediction accuracy following a hybrid fusion approach by which infrared spectra and XRF elemental contents are combined prior to modelling have ranged from consistently positive (for total C and N prediction [26]), to small and inconsistent (for soil texture prediction [17]), to non-existent (for texture, OC, total N, and pH [22]). Weindorf et al. [25] increased prediction accuracy for key properties of arid soils with another hybrid fusion approach that sequentially combined visNIR spectra and XRF elemental data, whereby penalized spline regression (PSR) using visNIR spectra was followed by random forest (RF) to fit XRF elemental data to the residuals of the PSR model. Finally, high-level fusion approaches, which combine predictions following separate multivariate statistical analysis, have been frequently applied to infrared and XRF data. Tested methods include, e.g., simple arithmetic averaging of predictions, variance weighted (VW) averaging, whereby the variance between estimates of repeated model runs affects the weight of a sensor in the model average [28], and Granger Ramanathan (GR) averaging, whereby a multiple linear regression (MLR) model is fit to the sensor predictions to determine the model average [29]. For the combined use of visNIR and XRF to predict a range of soil properties, O’Rourke et al. [18] found GR averaging had comparable or better performance than VW averaging and is also referred to the latter given its simplicity of calculation. High-level model averaging was very useful in their study, decreasing the prediction error of a regional Australian dataset by 23–29% for clay, sand, pH and CEC compared to the best single spectrometer, whereas Tavares et al. [27] found GR averaging of visNIR and XRF predictions improved prediction of some but not all tested soil fertility parameters compared to the best single spectrometer for a set of soils from two fields in Brazil. Additional systematic testing is required to determine the circumstances in which sensor fusion is most advantageous, considering both the prediction mechanisms of the sensors for the soil properties of interest and the methods of sensor fusion. Furthermore, the principle of parsimony (Occam’s Razor) should also be considered in choosing the optimal approach—i.e., model predictors should be limited to those whose removal would cause a significant increase in the error of estimation [30]. As a central purpose of implementing proximal sensors is to increase the time and cost efficiency of collecting high resolution soil information, sensor fusion is only recommended over the use of a single sensor if its leads to substantial improvement in model accuracy. Thus, model fusion studies must report the relative improvement in estimation accuracy resulting from model fusion compared to use of the best single spectrometer [20].

Given the need for improved MIR estimation accuracy for complex soil properties influenced by both soil organic and inorganic fractions, the lack of understanding of XRF prediction mechanisms, and the inconsistent usefulness of various model fusion approaches shown in past studies, the objective our of study was to systematically test separate and combined use of MIR and XRF for a wide range of soil properties and fusion approaches. Specifically, we investigate (i) the field-scale performance of models using MIR spectra and XRF elemental contents applied separately and in combination using several hybrid and high-level model fusion approaches for prediction of OC content (total and labile), total N content, texture, pH, and CEC; and (ii) the prediction mechanisms by which the XRF elemental contents can estimate the aforementioned organic and inorganic soil fractions, as well as the complex soil properties influenced by both. We hypothesized: (1) Compared to separate use of MIR, fusion of MIR and XRF worsens the estimation accuracy for properties related to the organic fraction (total OC and N content) since prediction mechanisms are more direct for MIR than XRF, with the former relying on spectrally-active bonds between light elements and the latter on covariations with heavier elements detected in the XRF range; (2) Estimation of properties related to the soil inorganic fraction (clay, silt, sand) is possible with either separate or joint use of MIR and XRF, as the bonds present in soil minerals can be detected by the former and elements dominating the soil matrix can be detected with the latter; and (3) Sensor fusion improves estimation accuracy most for complex properties with relatively indirect prediction mechanisms for both MIR and XRF (i.e., pH, labile OC content, and CEC) since the spectral ranges provide unique information that may increase model robustness. To test these hypotheses, we utilized an existing field-scale dataset [12] for which XRF measurements were additionally conducted. 

## 2. Materials and Methods

### 2.1. Field Sampling and Laboratory Analysis

The soil under investigation was an arable, silt loam Haplic Luvisol (16% clay, 80% silt, and 4% sand) in Lüttewitz (Saxony, Germany) [31]. This is a loess-derived soil, containing high-activity clays and high base saturation. The site has an elevation of 290 m, annual average temperature of 8.6 °C, and precipitation of 572 mm [32]. Management was consistent with standard agricultural practices, including conventional tillage with a moldboard plow to a depth of 30 cm. Sampling was conducted over five days in September 2016 as described in Greenberg et al. [12]. Sample points were laid out in a grid across a 52.5 m × 600 m field, resulting in a total of *n* = 120. *n* = 117 are included in the present study as three outlier sample units were identified and removed, as described below. At each sampling point, a 15 cm × 15 cm area was cleared of wheat stubble on the soil surface and soil was collected to a depth of 2 cm. 

Total C and N contents were analyzed on ball-milled soils by dry combustion with a CN elemental analyzer (Elementar Vario El, Heraeus, Hanau, Germany). Due to the absence of carbonates in the soil, total C was equivalent to total OC. Soil texture was determined with the pipette method according to DIN ISO 11277 [33]. The pH values were determined with 2.5 g field-moist soil in 6.25 mL 0.01 M CaCl_2_ according to DIN ISO 10390 [34]. For determination of CEC, the soil was first slowly leached with 0.1 M BaCl_2_, with a soil to solution ratio of 1:10. Next, exchangeable K^+^, Na^+^, Ca^2+^ and Mg^2+^ were measured in the filtered extracts with ion chromatography (850 Professional IC, 237 Metrohm, Herisau, Switzerland) and CEC was calculated as the sum of exchangeable cations [35]. Labile OC was separated by a modification of the method described by Zimmerman et al. [36]. For this, 15 g of soil was sonicated in 75 mL H_2_O (Branson Digital Sonifier, Branson Ultrasonics Corporation, Dietzenbach, Germany) at an energy level of 22 J mL^−1^ to break up macroaggregates. The soil was then wet-sieved using a 63-µm sieve, separating sand-sized particles from silt- and clay-sized particles. Deviating from the original method, dissolved OC was not collected from the suspension at this stage due to the small size of this fraction (2% of total OC for Zimmerman et al. [36]) and its heterogeneous chemical structure [37]. Analysis continued on the fraction >63 µm by separating particulate organic matter (POM) from sand and stable aggregates with 1.8 g cm^−3^ sodium polytungstate solution. This >63 µm POM represents the labile OC fraction [36,38], which was quantified based on mass recovery and the C content of the fraction determined as described above for total OC. Three outlier sample units were removed from the analysis because the distribution of OC among the fractions was starkly different than for other sample points and there was insufficient material to repeat the fractionation. Although these sample units were not outliers with regards to the other measured soil properties, they were eliminated from the datasets for all properties so that XRF prediction mechanisms for labile OC could be directly compared to those for other soil organic and inorganic properties utilizing an identical dataset.

### 2.2. Spectral Measurements

Prior to MIR and XRF measurements, soil was dried and ball-milled to <0.2 mm using a Retsch MM 400 (Haan, Germany) with 10 ZrO_2_ balls at 30 Hz for 5 min. For each spectral device, two replicate measurements per sample unit were taken and averaged. Specifications of MIR measurements are elaborated in Greenberg et al. [12]. Briefly, diffuse reflectance infrared Fourier transform spectra were measured with a Bruker-TENSOR 27 MIR spectrometer (Ettlingen, Germany) with an A562 integrating sphere detector and the diffuse-reflectance accessory (Ulbricht-Kugel, Ettlingen, Germany). The range 7000–4000 cm^−1^ (longwave NIR) was excluded from the analysis. The region <1000 cm^−1^ was also excluded due to limited usefulness caused by overlapping mineral and organic absorption bands [11]. Spectra were converted to absorbance (log_10_[1/reflectance]) for modelling.

X-ray fluorescence measurements were made with the bench-top Bruker S2 PUMA EDXRF spectrometer (Karlsruhe, Germany). This device has an Ag X-ray tube with a maximum power of 50 W, a HighSenseTM Silicon Drift Detector which can detect approximately 300,000 counts per second (cps), and a Peltier cooler. Measurements were made in He atmosphere with reduced pressure. A total of 4096 (12 bit) fluorescence intensities were recorded across energy channels from 0 to 44 keV. A quality check was performed at the beginning of each measurement day using a FLX-K04 glass standard to determine if a drift correction was required; however, no correction was required during the measurement campaign. Measurements were made with two voltage tube conditions: (i) 20 kV, 0.63 mA current, and 150 s spectral acquisition time with 25% dead time on average and (ii) 40 kV, 1.17 mA current, and 200 s spectral acquisition time with 39% dead time on average. Each sample contained 5 g of soil in a sample cup with a diameter of 3.6 cm and a mask size of 3.4 cm. Cups were sealed with a SpectroMembrane^®^ Prolene^®^ Thin-Film with 4.0 µm thickness (Chemplex Industries, Inc., Palm City, FL, USA) and rotated during measurement.

**Figure 1:**
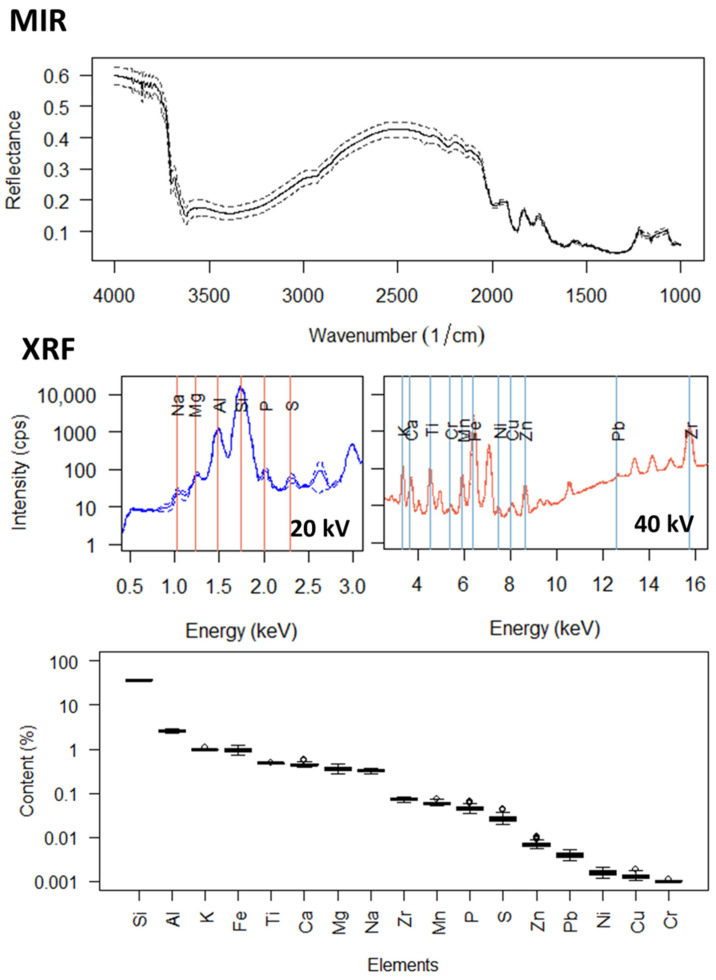
Average (solid lines) ± 2 standard deviations (dotted lines) mid-infrared (MIR) and X-ray fluorescence (XRF) spectra for *n* = 117 soils, as well as boxplots of the total elemental contents estimated by XRF. Kα_1_ lines for each element are shown, besides for Pb, which utilized the Lβ_1_ line. The y-axes of the XRF spectra and elemental contents have logarithmic scales.

The XRF approach utilized in this study was to determine total elemental contents by spectral deconvolution rather than making evaluations directly with the XRF spectra. Spectral deconvolution is considered an optimized data evaluation technique for XRF due to the complexity of physicochemical relationships, which create a risk of poor model outcomes due to spectral artifacts arising from the X-ray tube source, but also artifact peaks arising from the detection process [13,39]. An internal, field-scale calibration for the XRF elemental predictions was performed with the Bruker AXS SPECTRA.ELEMENTS software (version 2.4, Bruker AXS GmbH, 2020) using total elemental contents determined by reference methods for *n* = 15 soils. This is in contrast to the approaches of many recent XRF studies predicting soil properties, which used only generic, factory-installed calibrations designed for measurement of soils [18,19,20,24,26,40,41]. The sample units used for internal calibration were selected to cover the variability in CEC existing in the field. The reference values for total elemental contents were obtained by acid digestion followed by Inductively Coupled Plasma Optical Emission Spectroscopy (ICP-OES). For this, soils were dried at 40 °C and ball-milled to <0.2 mm using a Retsch MM 400 (Haan, Germany) with 10 ZrO_2_ balls at 30 Hz for 5 min. 50 mg of soil were fully digested at high pressure with a mixture of ultrapure concentrated HNO_3_, HCl and HClO_4_ and HF in closed pre-cleaned PTFE vessels using a PicoTrace DAS 30 system. This technique was previously shown to achieve total dissolution of soil samples, including refractory phases [42]. The reproducibility of the element measurements is better for completely digested samples than partially digested samples: for completely digested samples, the elemental concentrations are independent of the interaction time between the sample and acids, while for partially digested samples, the elemental concentrations correlate with the duration of the digestion [43,44], and therefore differ depending on the precise methodology applied. The sample solutions were then analyzed by ICP-OES using an Agilent 5100 VDV spectrometer with an inductively coupled Ar plasma as the source, which was calibrated with a standard soil solution. Si cannot be measured using ICP-OES due to loss during acid evaporation. Si contents for the calibration subset were therefore obtained via Wavelength Dispersive XRF (WDXRF) on glass disks using a Malvern Panalytical Axios advanced spectrometer (Rh X-ray tube) and the software SuperQ (version 4) at the University of Göttingen. For this, measurements were made on glass disks containing 0.42 g soil mixed with 4.2 g of A12 flux (66% di-lithium tetraborate and 34% lithium metaborate). Si was measured using a PE 002C crystal, a 550 µm collimator and a flow detector. Measurements (60 s counting time) were performed at a peak angle of 109.0258 (2θ°) and with a voltage of 25 kV and a current of 160 mA. Three soil standards covering the SiO_2_ range of the investigated samples as well as JR-1, a GSJ international rock standard with similar SiO_2_, were processed and measured with the sample set. For all standards, SiO_2_ was within 3% of the accepted values [45]. In the internal calibration, 17 elements had concentrations above the detection limit. The R^2^ between reference (WDXRF for Si and ICP-OES for all other elements) and EDXRF elemental contents was greater than 0.8 for Si, Al, K, Fe, Ca, Mg, S, Zn, Ni, and Cu, ranged from 0.5–0.8 for Ti, Na, Mn, P, and were less than 0.5 for Cr, Zr, and Pb. These 17 element concentrations were then predicted from the XRF 20 kV spectra for lighter elements (Na, Mg, Al, Si, P, S) or the 40 kV spectra for heavier elements (K, Ca, Cr, Mn, Fe, Ni, Cu, Zn, Pb) using the Bruker AXS SPECTRA.ELEMENTS for all *n* = 117 soils. Figure 1 shows the MIR and XRF spectra, as well as boxplots of the total elemental contents estimated from the XRF spectra.

### 2.3. Separate Modelling of MIR and XRF

Prior to modelling the soil properties of interest, the field-scale dataset was divided into spatially-separated thirds and a three-fold training/testing procedure was carried out using 2/3 of the sample in training (*n* = 78), which included leave-one-out cross-validation, and 1/3 of the sample in validation (*n* = 39). This procedure was repeated three times until each of the spatially-separated folds of the dataset was used for validation once.

The objective of modelling was to predict contents of total and labile OC, total N, clay, silt, and sand, as well as pH and CEC. Separate modelling was carried out with two types of predictors: MIR absorbances (1555 wavelengths) and XRF elements (17 elements). Analysis was performed with the statistical software R (version 4.1.1, R Core Team, 2021). The modelling approach was partial least squares regression (PLSR) with the ‘pls’ package [46], which was selected due to its ability to handle collinearity between predictors and maximize the explained variance between predictors and response variables while eliminating noise [47]. For modelling with XRF elements, we also performed an RF analysis and MLR; however, only results of PLSR are presented since they were consistently superior. The XRF elemental data were z-transformed prior to PLSR and subsequent VIP analysis described below. For MIR data, 13 spectral pretreatments were carried out with the ‘prospectr’ package [48], including (i) use of the full spectra without manipulation, (ii–v) calculation of moving averages (calculated over 5, 11, 17 or 23 data points), and (vi–xiii) application of the Savitzky-Golay algorithm for the reduction of noise applied with the polynomial degree (PD) set to 2, the order of the derivative (DER) ranging from 1 to 2 (with PD-DER: 2–1 or 2–2), and a window smoothing size of 5, 11, 17 or 23.

To determine the optimal number of latent variables for MIR and XRF, model training included leave-one-out cross-validation for each of the three training folds. The maximum number of latent variables was set to 15. To create more robust, parsimonious models, the optimal number of latent variables was determined in cross-validation by considering minimization of Akaike Information Criterion (AIC) [49] calculated as:

$$\mathrm{AIC}=n\times \log_{e}\left(\mathrm{RMSE}\right)+2k$$

where *n* is the sample size, *k* is the number of latent variables, and RMSE is calculated as:

$$\mathrm{RMSE}=\;\sqrt{\frac{\sum^{\;}{\left({\hat{y}}_{i}-y_{i}\right)}^{2}}{n}}$$

where ${\hat{y}}_{i}$ is the modelled soil property, $y_{i}$ is the measured soil property, and *n* is the sample size. For MIR data, the optimal spectral pretreatment was the model with the highest ratio of performance to interquartile distance (RPIQ) in cross-validation, calculated as:

$$\mathrm{RPIQ}\;=\frac{\mathrm{IQR}}{\mathrm{RMSE}}$$

where IQR is the interquartile range of the measured soil property. RPIQ was calculated rather than ratio of prediction to deviation (RPD) due to non-normality of contents of total and labile OC, total N, clay, silt, and sand according to the Shapiro-Wilk test. Results were evaluated according to the classification system of Chang et al. [50]. For this, the RPD classification system was converted to RPIQ values. For a normally-distributed variable and large sample size, RPIQ = 1.89 corresponds to RPD = 1.4 and R^2^ = 0.5. Thus, a model with RPIQ < 1.89 is considered poor, 1.89–2.70 is satisfactory, and >2.70 is very good. However, one has to keep in mind that the usefulness of a model must always be judged based on the context in which it is applied. The optimal PLSR models generated in training for the three dataset partitions were then tested with the validation sets.

### 2.4. Model Fusion Approaches

Four fusion variants were carried out, as described below and summarized in Table 1. The first two variants can be considered hybrid approaches, since XRF spectra were first used to estimate total elements contents, and then these contents were combined with information from the MIR range. Both hybrid approaches followed a sequential fusion procedure, which began with MIR-PLSR prediction of soil properties as described above. Subsequently, one approach, hereafter referred to as MIR-XRF_HYB1_, used a concatenated matrix of the MIR estimate for a given soil property and all XRF elemental contents (i.e., 18 total predictors) to carry out PLSR with the model training and testing approach described above. The other sequential approach, hereafter referred to as MIR-XRF_HYB2_, involved prediction of the residuals of MIR-PLSR using XRF elemental contents for PLSR, followed by summation of the MIR estimates of the soil properties and XRF estimates of the residuals, similar to the sequential approaches of Towett et al. [22], Weindorf et al. [25] and Chakraborty et al. [51].

Finally, two high-level fusion approaches were tested, including (i) arithmetic (MIR-XRF_AVG_) and (ii) Granger-Ramanathan averaging (MIR-XRF_GR_) [29] of the MIR-PLSR and XRF-PLSR estimates after separate modelling. The latter approach fits an MLR to the measured lab data and the predicted values from the two sensors for the soil property of interest as follows:

y=xβ+e

where *y* is the vector of measured values, *x* is the matrix of predictions (i.e., the separate predictions of MIR-PLSR and XRF-PLSR). Ordinary Least Squares is then used to solve for the intercept (*e*) and the vector of weights of each prediction (*β*). The regression models were created with the training sets and then applied to the test sets. Calculations were carried out with the R package ‘GeomComb’ and the function comb_OLS() [52].

In order to determine the usefulness of model fusion, we calculated the relative improvement in estimation accuracy resulting from model fusion, expressed as the percentage increase in RPIQV from the best single spectrometer to the best fusion approach.

### 2.5. Evaluation of XRF Prediction Mechanisms

The relative importance of the XRF element predictors in the PLSR models described above was evaluated for each response variable by calculating the variable importance in the projection (VIP) for element *j* as:

$${\mathrm{VIP}}_{j}=\sqrt{p\sum_{a=1}^{A}\left[SS_{a}{\left(\frac{w_{aj}}{||w_{a}||}\right)}^{2}\right]/\sum_{a=1}^{A}\left(SS_{a}\right)}$$

where *p* is the total number of predictors (i.e., elements), *SS_a_* is the sum of squares explained by the *a*th component, and ($w_{aj}/||w_{a}||$)^2^ is the normalized importance of the *a*th component of the *j*th element [53]. This analysis was carried out using the VIP() function of the ‘plsVarSel’ package [54]. For each response variable, VIP scores for element predictors were calculated for each of the models resulting from the dataset partitions and then averaged. As the average of all squared VIP scores for a response variable equals 1 [55], comparison of VIP scores uses a threshold of 1 to identify important element predictors for each soil property. Relationships between the soil properties and their important element predictors were inspected with scatterplots and Spearman rank correlations.

## 3. Results and Discussion

### 3.1. Prediction of Properties Related to the Organic Soil Fraction

**Figure 2:**
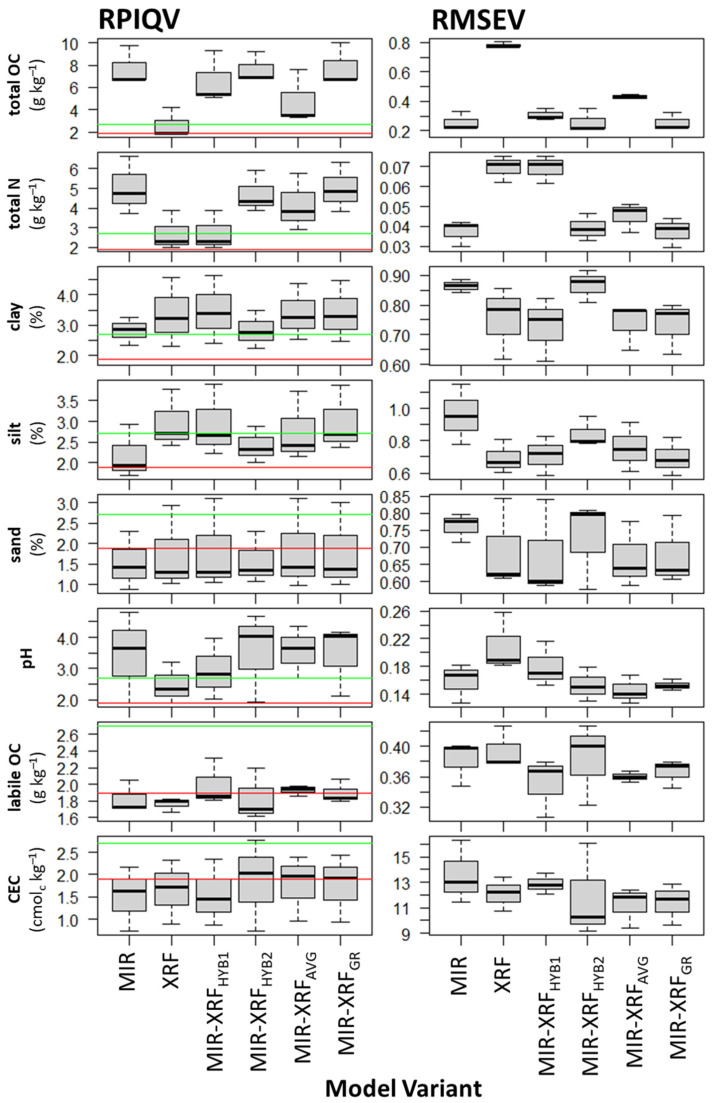
Validation RPIQ and RMSE for the model variants across the three dataset partitions. RPIQV values below the red lines refer to poor model accuracy (<1.89) and values on and above the green lines refer to very good model accuracy (≥2.70). See Table 1 for definitions of model variants.

**Figure 3:**
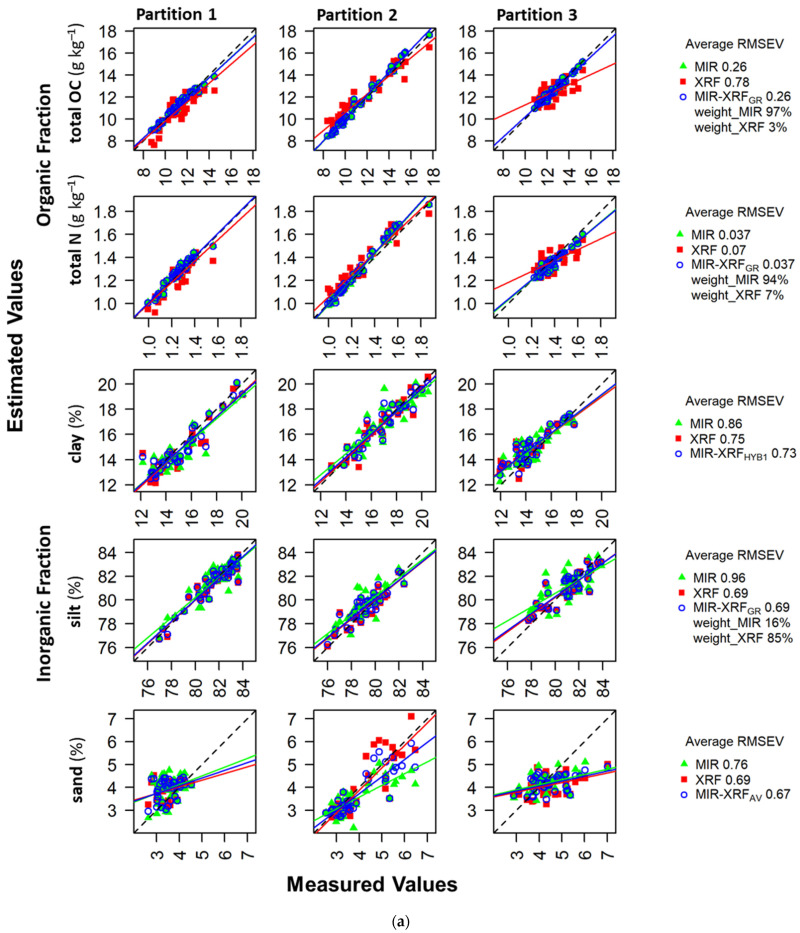

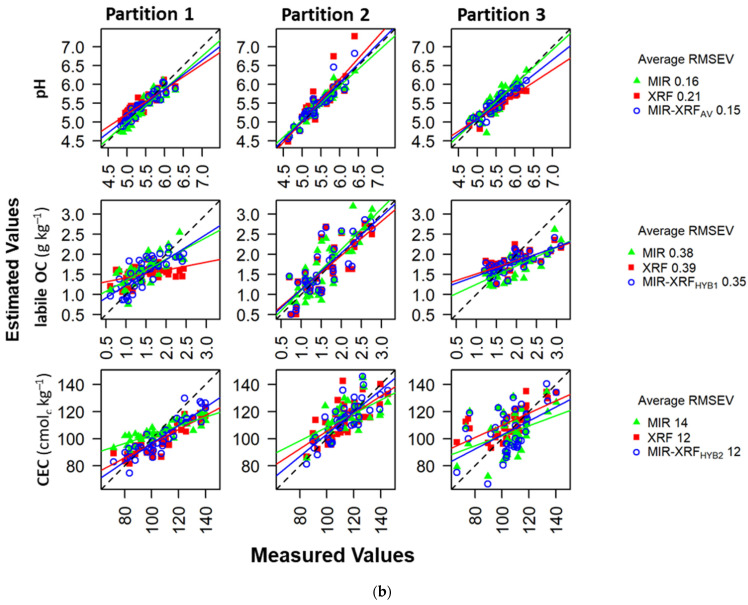
Measured values vs. validation estimates for (**a**) total OC, total N, clay, silt and sand content and (**b**) pH, labile OC content, and CEC using single spectrometers and the best fusion approach according to average RPIQV. Data include validation estimates for all three partitions of the dataset, average RMSEV across the partitions, and weights of the spectrometers in MIR-XRF_GR_ approach where applicable. Regression lines are fit to the data to observe bias. See Table 1 for definitions of model variants.

We hypothesized that compared to separate use of MIR, fusion of MIR and XRF worsens the estimation accuracy for properties related to the organic fraction since prediction mechanisms are more direct for MIR than XRF, with the former relying on spectrally-active bonds between light elements and the latter on covariations with heavier elements detected by XRF. For total OC and N, MIR greatly outperformed XRF in both model training and testing (Table 2). MIR and XRF had an average RPIQV of 7.72 and 2.66, respectively, for total OC and 5.04 and 2.71 for total N. MIR prediction of total OC and N was very good across all dataset partitions, whereas XRF performance ranged from satisfactory to very good (Figure 2). For total OC, fusion approaches ranged from severely worsening performance to slightly improving performance (MIR-XRF_GR_ average RPIQV = 7.82) compared to MIR (Table 2). For total N, fusion approaches ranged from severely to very slightly (MIR-XRF_GR_ average RPIQV = 4.99) worsening performance compared to MIR (Table 2). MIR-XRF_GR_ was the optimal fusion approach in model training and testing for both total OC and N. Notably, the average weighting of MIR in the MIR-XRF_GR_ was 97% and 94% for total OC and N, respectively—thus, MIR predictions dominated this fusion approach, which can be seen from the similarity of the MIR and MIR-XRF_GR_ estimates (Figure 3a).

Other studies conducted at larger scales likewise found superior performance of MIR over XRF for prediction of properties related to the organic fraction. Using a regional dataset of Anthrosols in China, Xu et al. [19] found good prediction of SOM and total N (RPIQV > 2.5) with MIR using a genetic algorithm (GA) combined with PLSR, whereas performance was poor with XRF spectra and GA-PLSR, and there was also no benefit of low-level data fusion compared to MIR. For a national Irish dataset, O’Rourke et al. [20] also found better validation performance for MIR than XRF spectra for prediction of OC content (R^2^ = 0.78 vs. 0.70) and no improvement through GR averaging of predictions (R^2^ = 0.78). Finally, using soils across sub-Saharan Africa, Towett et al. [22] found prediction of OC and total N with MIR-RF was superior (R^2^ = 0.90 and 0.86, respectively) to RF with XRF spectra (R^2^ = 0.68 and 0.63, respectively), and that concatenation of MIR and XRF spectra matched performance of MIR.

**Figure 4:**
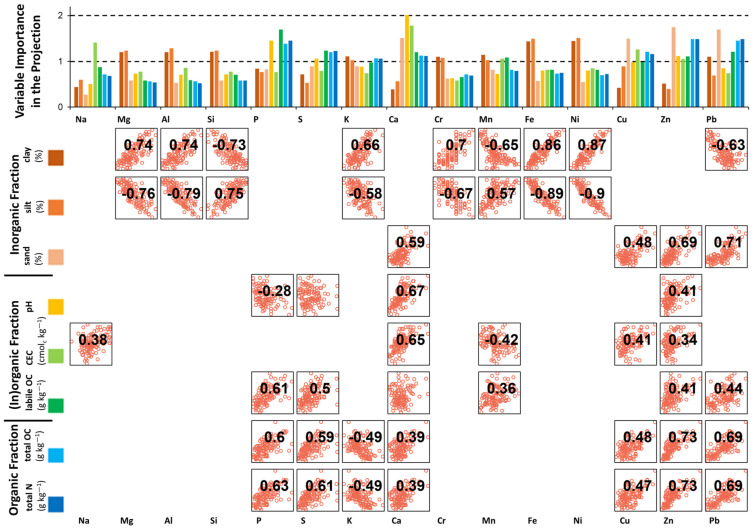
Average variable importance in the projection (VIP) scores for XRF element predictors in PLSR models to estimate soil properties. A threshold at VIP = 1 marks the average of all squared scores [55]. Scatterplots are shown for soil properties and XRF elements with average VIP > 1, and Spearman rank correlation coefficients are given for pairs with significant (*p* < 0.05) correlations. Ti and Zr had no VIP > 1 and therefore are not shown for simplicity.

The MIR prediction mechanisms for total OC and N rely on fundamental vibrations of compounds contained in soil OM, e.g., alkyl groups (-CH_2_) at 2930–2850 cm^−1^, protein amide at 1670 cm^−1^, and aromatic groups from 1600–1570 cm^−1^ [7]. For our XRF models, predictions relied on covariations of OC and N with heavier elements that could be quantified by the Bruker S2 Puma (Z > 10). VIP analysis found the same important element predictors for both total OC and N (Figure 4). Using a threshold of VIP = 1, the important elements were Zn ≈ Pb > P > Cu ≈ S > Ca > K—all with positive correlations with total OC and N, with the exception of a negative correlation with K. Spearman rank correlations with VIP > 1 elements were all significant (*p* < 0.05) and ranged from ±0.39 > r_s_ > ±0.73. Given that >90% of total soil N is composed of organic N [56], and plants and microorganisms require N in set proportions to C, the similarity of the prediction mechanisms for OC and N content is expected. Contents of Zn and Cu, both plant micronutrients, have been found to correlate with SOM content [57,58]. Cu and, to a lesser extent, Zn cations react with organic compounds to form water-insoluble complexes [59]. Pb bioaccumulates in organisms over time [60], which could explain its positive relationship with OC and N. The correlations with P and S could be due to the high proportions of total P (20–80%) and S (>90%) stored in organic forms in soil [58]. Silva et al. [61] likewise found great importance of Zn and P as XRF predictors of SOM content for Brazilian Cambisols, and O’Rourke et al. [20] also found a strong Pearson correlation (r = 0.82) between S and OC for diverse soils from a national Irish dataset. The inverse relationship of K with OC and N could relate to the importance of K for predicting clay content (discussed subsequently), and the negative correlation of total OC and clay content at this site (r_s_ = −0.48, *p* < 0.01).

It has to be noted that XRF spectra have previously been used to predict properties which are spectrally inactive for this approach. For instance, Morona et al. [21] used XRF spectra in combination with PLS regression for a prediction of OC contents, although it is well known that this relies on a highly indirect prediction approach. Moreover, the XRF spectra contain not only artifact peaks arising from the X-ray tube source, but also artifact peaks arising from the detection process [13,39]. For an optimal data evaluation, these artifacts need to be appropriately accounted for in spectral deconvolution [13] rather than just using simple multivariate statistical approaches. The use of the elemental concentrations obtained in high accuracy with XRF for a prediction of soil properties of interest using indirect relationships emphasizes that for any spectroscopic approach applied, the fundamental chemical relationships should not be disregarded and applications outside the spectrally-active properties should be done cautiously. Overall, an accurate prediction of organic properties with XRF cannot be expected for any site or region unless close indirect correlations between the elemental contents responsive to XRF and organic properties such as the OC content exist. Quantification of such correlations sheds light on the underlying processes, which would not be possible if just the raw spectra were used. Therefore, although our approach required an additional calibration of the elemental contents to the spectra, the results are more enlightening in terms of understanding the prediction mechanisms for XRF.

In summary, although the indirect prediction mechanisms for XRF estimation of total OC and N were useful at the field-scale, the accuracy of MIR, relying on more direct prediction mechanisms, was clearly superior. Our hypothesis—that sensor fusion could worsen performance for properties related to the organic fraction—was partially supported: fusion approaches ranged from roughly matching to severely worsening the superior performance of MIR. Of the fusion approaches tested, GR averaging bears mentioning, as its validation performance was very similar for total N and OC to that of MIR. Since this high-level fusion approach gives weights to the predictions according to the accuracy of each sensor in model training, the more useful spectrometer will be given a dominant weight in the average. Thus, this is a robust approach to capturing the potential benefits of multiple sensors without risking substantially worsening performance compared to the best single spectrometer.

### 3.2. Prediction of Properties Related to the Inorganic Soil Fraction

We hypothesized that estimation of properties related to the soil inorganic fraction is possible with either separate or joint use of MIR and XRF, as the bonds present in soil minerals can be detected by the former and elements dominating the soil matrix can be detected with the latter. For clay, silt and sand content prediction, XRF outperformed MIR in both model training and testing (average clay RPIQV of 3.37 and 2.82, silt RPIQV of 2.97 and 2.18, and sand RPIQV of 1.75 and 1.53, respectively). In validation, clay prediction across dataset partitions ranged from very good to satisfactory for both XRF and MIR, silt prediction ranged from very good to satisfactory for XRF and very good to poor for MIR, and sand prediction range from very good to poor for XRF and satisfactory to poor for MIR. The low variability of sand content at the site (2.5–7.0%, Figure 3a) explains the low RPIQV values, as RMSEV values were comparable across all size fractions (Figure 2).

For prediction of clay content in validation, fusion approaches ranged from roughly matching the inferior performance of MIR to marginally improving upon the superior performance of XRF (average RPIQV = 3.47 for MIR-XRF_HYB1_). It is notable that the inferior fusion approach (MIR-XRF_HYB2_) is MIR dominant, with XRF only predicting the variance in clay not explained by MIR, whereas the superior fusion approach (MIR-XRF_HYB1_) is dominated by XRF predictors (17 XRF elements vs. 1 MIR prediction). For prediction of silt content in validation, fusion approaches ranged from falling between the performance of MIR and XRF to performing similarly to XRF (MIR-XRF_GR_ RPIQV = 2.98). Again, an MIR-dominant fusion approach (i.e., MIR-XRF_HYB2_) was inferior to an XRF-dominant approach (MIR-XRF_GR_ with 85% weighting for the XRF estimate). For prediction of sand content in validation, fusion approaches ranged from falling between MIR and XRF performance to slightly outperforming XRF (MIR-XRF_AV_ RPIQV = 1.83). MIR-XRF_GR_ was the optimal fusion approach for all texture classes in model training, but this only remained the case in validation for silt.

Other studies have also investigated the ability of XRF and infrared spectrometers, alone or in tandem, to predict soil texture classes at larger spatial scales. Wang et al. [17] found for a set of spiked regional calibrations validated with two district-level test sets in China that XRF accuracy was as good or better than NIR for validation prediction of clay, silt, and sand fractions, and that a sequential fusion approach improved predictions in some cases, but with a higher weighting of XRF element predictors compared to principal components calculated from the NIR spectra. O’Rourke et al. [18] predicted clay and sand with XRF (both with spectra using a cubist algorithm and elements with stepwise MLR), visNIR (spectra with cubist algorithm), as well as the GR average of the two spectral estimates for a regional Australian dataset with 25% of sample units reserved for validation. For clay validation, they found comparable performance of XRF and visNIR and benefits of high-level model fusion: XRF(spectra)-cubist (R^2^ = 0.77) < visNIR-cubist (0.86) ≈ XRF(elements)-stepwise MLR (0.86) < GR averaging (0.93). For sand validation, they found superior performance of XRF, but nevertheless a benefit of high-level model fusion: visNIR-cubist (R^2^ = 0.35) < XRF(spectra)-cubist (0.79) ≈ XRF(elements)-stepwise MLR (0.80) < GR averaging (0.85). In contrast, Towett et al. [22] found for a set of *n* = 700 soils across sub-Saharan Africa, with 30% of sample units reserved for validation, that prediction of clay, silt, and sand with MIR-RF was slightly to somewhat superior (0.60 < R^2^ < 0.74) to RF with XRF spectra (0.49 < R^2^ < 0.73), and that concatenation of predictors roughly matched performance of MIR.

For MIR, prediction of soil texture relies on vibrations of compounds within clay minerals of the clay-sized fraction (<2 µm), e.g., smectite and illite (3630–3620 and 3400–3300 cm^−1^), and vibrations of quartz (1100–1000 cm^−1^) for the sand-sized fraction (2000–63 µm) [7]. Prediction mechanisms for the silt-sized fraction (63–2 µm) are less clear, as the minerals composing this fraction may be more variable, potentially containing quartz, primary silicate minerals (e.g., muscovite), secondary silicate minerals (e.g., smectite), as well as other secondary minerals (e.g., Fe- and Al-oxides) [62].

For XRF prediction of clay, nine elements had an average VIP > 1, including in order of decreasing importance and with associated significant correlation coefficients, Fe (r_s_ = 0.86) ≈ Ni (0.87) > Si (−0.73) ≈ Al (0.74) ≈ Mg (0.74) > Mn (−0.65) ≈ K (0.66) ≈ Pb (−0.63) ≈ Cr (0.70) (Figure 4). Si, Al, and Mg are important components of aluminosilicate clay minerals, with isomorphic substitution incorporating Fe into the structure, and K occupying interlayer and exchange sites [58]. For XRF prediction of silt, eight elements had an average VIP > 1, including in order of decreasing importance Fe ≈ Ni > Al ≈ Si ≈ Mg > Cr ≈ K ≈ Mn, which all had significant and similarly strong correlations with silt content. This overlaps substantially with the important element predictors for clay, except that the directions of relationships were opposite (Figure 4), which suggests silt content was predicted, firstly, by the absence of key elements in clay, and secondly, by the higher Si content in silt, due to the presence of, e.g., quartz (SiO_2_). For XRF prediction of sand, four elements had an average VIP > 1, including in order of decreasing importance Zn (r_s_ = 0.69) > Pb (0.71) > Ca (0.59) ≈ Cu (0.48). Though all correlations were significant, the relationships were weaker than for clay and silt. There may have been indirect prediction of sand based on a positive correlation with total OC content (r_s_ = 0.56, *p* < 0.01), which likewise positively correlated with Zn, Pb, Ca and Cu. Zhu et al. [40], who also predicted soil texture with XRF elemental data, noted the great dependence of the relationships between elemental contents and soil texture on soil parent material and geophysical setting (e.g., degree of weathering). However, their study, conducted on highly varied soil types, found results to suggest a degree of at least regional stability to the clay:Fe relationship. This is corroborated by results of Tavares et al. [23] and O’Rourke et al. [18], who likewise found a high dependency between Fe and clay content for larger-scale datasets.

Returning to our hypothesis—estimation of clay, silt, sand is possible with either separate or joint use of MIR and XRF—we can confirm that while both MIR and XRF alone or together could achieve satisfactory to very good clay estimations for all dataset partitions, only XRF or joint analysis could achieve this for silt, and all approaches had poor sand prediction according to RPIQV due perhaps to the low sand variability present. At the field scale, XRF and XRF-dominant fusion approaches were superior to MIR for predicting soil texture classes. Future research should continue to explore the usefulness of XRF versus infrared and fused approaches at larger spatial scales, with particular emphasis on designing calibrations that are either robust to differences in soil parent material and geophysical setting or manage to stratify soils into suitable sets for modelling.

### 3.3. Prediction of Properties Affected by Both the Organic and Inorganic Soil Fraction

We hypothesized that sensor fusion improves estimation accuracy most for properties with relatively indirect prediction mechanisms for both MIR and XRF since the spectral ranges provide unique information that may increase model robustness.

For pH, MIR greatly outperformed XRF in model training and testing (RPIQV of 3.45 and 2.49, respectively) (Table 2). While MIR-XRF_GR_ was the optimal fusion approach in training, MIR-XRF_AV_ was superior in validation. Fusion approaches ranged from falling between the performance of XRF and MIR to marginally improving upon the superior performance of MIR (MIR-XRF_AV_ RPIQV = 3.56). In validation, pH prediction across the dataset partitions ranged from very good to satisfactory for both XRF and MIR, while MIR-XRF_AV_ achieved very good performance for all partitions.

Other studies conducted at larger scales have likewise found superior performance of MIR over XRF for prediction of pH. Using a regional dataset of Anthrosols in China, Xu et al. [19] found good prediction of pH (RPIQV > 2.5) with MIR using GA-PLSR, whereas performance was poor with use of XRF spectra and GA-PLSR. For a national Irish dataset, O’Rourke et al. [20] also found better validation performance for MIR than XRF spectra with a cubist algorithm for prediction of pH (R^2^ = 0.83 vs. 0.51), and small further improvement through GR averaging of XRF, visNIR and MIR predictions (R^2^ = 0.86). Using a dataset of soils across sub-Saharan Africa, Towett et al. [22] found prediction of pH with MIR-RF was superior (R^2^ = 0.82) to RF with XRF spectra (R^2^ = 74, respectively), and concatenation of MIR and XRF predictors matched performance of MIR.

MIR prediction of pH relies on spectrally-active properties, such as CaCO_3_ content (which was not present in this soil) as well as organic molecules and clay minerals [7] due to their pH buffering effects but also their influence on acidifying biological processes [62]. For XRF prediction of pH, four elements had an average VIP > 1, with high importance of Ca (r_s_ = 0.67), followed by P (−0.28), Zn (0.41) and S (not significant). Although the soil did not contain CaCO_3_, total Ca content could indicate the ability of Ca-containing minerals to contribute to base saturation as they weather, buffering soil acidity [63]. Tavares et al. [23] and Silva et al. [61] likewise found Ca was the most important element predictor for pH for Brazilian Lixisols, Ferrasols and Cambisols. P, S and Zn were also important predictors for organic soil fractions (total OC and N), which are known to influence soil pH.

For labile OC, MIR slightly outperformed XRF in model training and testing (RPIQV of 1.83 and 1.76, respectively). While MIR-XRF_GR_ was the optimal approach in training, the sequential MIR-XRF_HYB1_ was superior in validation. Fusion approaches ranged from roughly matching to improving upon the superior performance of MIR (MIR-XRF_HYB1_ RPIQV = 2.00). In validation, labile OC predictions were poor for all partitions with XRF, and ranged from poor to satisfactory with MIR, as well as all sequential and high-level fusion approaches.

For MIR, labile OC prediction mechanisms may utilize unique spectral signatures of labile OC compounds (e.g., aliphatic peaks around 3000–2800 cm^−1^ [11]) as well as rely on indirect estimations based on relationships with total OC and clay minerals [12,64]. For XRF prediction of labile OC, six elements had an average VIP > 1, including in order of decreasing importance, P (r_s_ = 0.61) > S (0.50) ≈ Pb (0.44) ≈ Ca (not significant) > Zn (0.41) > Mn (0.36). This overlaps substantially with the important predictors for OC and N content, but with a greater usefulness of P for labile OC compared to Zn and Pb for total OC.

For CEC, XRF slightly outperformed MIR in training and testing (RPIQV of 1.65 and 1.51, respectively). While MIR-XRF_GR_ was the optimal approach in training, MIR-XRF_HYB2_ was superior in validation. Fusion approaches ranged from falling between the performances of MIR and XRF to outperforming XRF (MIR-XRF_HYB2_ RPIQV = 1.85). Figure 3b demonstrates how this sequential fusion approach corrected bias in the estimations of MIR and XRF for high and low CEC values. While performance for one partition was poor across all approaches, the sequential MIR-XRF_HYB2_ improved performance for the other partitions from poor to satisfactory and satisfactory to very good.

Several other studies have also tested the usefulness of XRF for estimating CEC. Sharma et al. [41] predicted CEC for *n* = 360 soils from two sites with diverse texture with XRF elements and stepwise MLR and found superior performance in model training (RMSE = 2.5 cmol_c_ kg^−1^) compared to the present study (RMSE = 10.4 cmol_c_ kg^−1^), perhaps due to their larger training set and inclusion of multiple soils from the same profiles. Tavares et al. [23] also successfully estimated CEC with 10 XRF emission peaks measured using MLR for *n* = 102 soils from two sites in Brazil, with 30% of sample units reserved for validation, resulting in RPIQV = 3.39 (converted from RPDV). Finally, O’Rourke et al. [18] predicted CEC with XRF (both with spectra using cubist and elements with stepwise MLR), visNIR (spectra with cubist), as well as the GR average of the two spectral-cubist estimates for a regional Australian dataset with 25% of sample units reserved for validation. They found a performance ranking in validation of visNIR-cubist (R^2^ = 0.44) < XRF(elements)-stepwise MLR (0.61) < XRF(spectra)-cubist (0.78) < GR averaging (0.81). Thus, as in the current study, they found superior performance of XRF over infrared spectroscopy, but also a benefit of data fusion.

CEC is affected by both the amount and exchange capacity of SOM and clay colloids present in soil [62]. Thus, there are several possible indirect prediction mechanisms for MIR and XRF. For MIR, signatures of organic compounds and clay minerals may be useful, but also absorbances at 2450 and 3350 cm^−1^ by interstitial water molecules surrounding interlayer cations within 2:1 clays, which have notably high CEC [7]. For XRF prediction of CEC, five elements had an average VIP > 1, including in order of decreasing importance, Ca (r_s_ = 0.65) > Na (0.38) > Cu (0.41) > Mn (−042) ≈ Zn (0.34). Tavares et al. [23] and Silva et al. [61] likewise found that Ca was the most important predictor for CEC for Brazilian Lixisols, Ferrasols and Cambisols. Ca and Na are cations generated by weathering of primary minerals and retained in the soil by CEC [63]. CEC shared important element predictors with total OC (Ca, Cu and Zn), pH (Ca and Zn), and clay (Mn). For this dataset, there was no significant correlation between total OC and CEC, but there were significant positive correlations between CEC and clay content (r_s_ = 0.35) as well as pH (r_s_ = 0.52). Thus, CEC may be mostly supplied by the high-activity clay minerals in this Haplic Luvisol, with meaningful increases to CEC from pH-dependent charges [58].

In summary, our hypothesis—that sensor fusion improves estimation accuracy most for properties with relatively indirect prediction mechanisms for both MIR and XRF—was partially supported. The relative improvement in estimation accuracy resulting from model fusion, expressed as the percentage increase in RPIQV from the best single spectrometer to the best fusion approach, was only 3.2% for pH (Table 2). This is similar in scale to the relative improvement through model fusion for clay (3.1%), silt (0.3%), sand (4.6%), total OC (1.3%), and total N (−1%, due to a worsening of performance through fusion) (Table 2). The relative improvement to estimation accuracy from model fusion was greater for labile OC (9.3%) and CEC (12.1%).

### 3.4. Comparison of Model Fusion Approaches

While the same single spectrometer was always optimal in both model training and testing, the optimal fusion approach often differed. In fact, each of the four tested fusion approaches was optimal in validation for at least one property. This points to the relatively minor differences in performance between some fusion approaches which utilized different fusion mechanisms, yet had similar dominance of one spectrometer over the other. More concretely, fusion approaches for which MIR was dominant were more useful for properties related to the organic fraction, while XRF-dominant approaches were more useful for properties related to the inorganic fraction. Tavares et al. [27] likewise found that for six tested fusion approaches combining visNIR and XRF sensors, no approach was consistently optimal, concluding that selection of the fusion approach must be attribute-specific. Thus, understanding the spectral prediction mechanisms for a soil property (i.e., the strength of the relationships between the property of interest and spectrally-active properties) should serve as a guide to selecting a suitable approach—especially given that model fusion worsened performance if the suboptimal sensor was dominant. The similar findings of Javadi et al. [65]—that fusion of visNIR and XRF sensors can worsen prediction accuracy in the case that one sensor greatly outperforms another—also demonstrate the need for strategic implementation of multi-sensor fusion. For applications where little is known about the prediction mechanisms, GR averaging can be recommended as a low-risk approach to capturing the potential benefits of multiple sensors without the need for exhaustive testing.

## 4. Conclusions

For this field-scale loess site, the optimal sensor for predicting properties related to the organic fraction (total OC and N contents) was MIR, while the optimal sensor for predicting properties related to the inorganic fraction (clay, silt and sand contents) was XRF. The benefits of the optimal model fusion approach were marginal to non-existent for these properties, and thus, according to the principle of parsimony, use of the best single spectrometer is recommended. Investigation of XRF prediction mechanisms found clay and silt contents had a large number of elements with VIP scores > 1 with strong correlations with clay and silt, explaining their superior XRF estimation accuracy compared to other properties. For complex soil properties affected by both organic and inorganic soil fractions, the benefit of the optimal model fusion approach compared to the best single spectrometer were marginal for pH, but more pronounced for labile OC and CEC. Thus, model fusion can be most recommended for labile OC and CEC. As dominance of a suboptimal spectrometer in a fusion approach worsened performance compared to the best single spectrometer, model fusion approaches should be applied with knowledge of the spectral prediction mechanisms. Without this, GR averaging can be recommended as the most robust approach to capturing the potential benefits of multiple sensors.

Future studies should determine if the findings of this field-scale case study also hold true at larger spatial scales. Additionally, systematic testing is required for in-situ MIR and XRF measurements, as the presence of soil moisture, coarse particles, and surface roughness may shift the relative advantages of the individual and combined use of these sensors.

## Figures and Tables

**Table 1:** Names and descriptions of modelling approaches. PLSR = partial least squares regression.

Variant	Descriptions
MIR	PLSR with 1555 MIR absorbances in the range 4000–1000 cm^−1^.
2. XRF	PLSR using 17 total elemental contents estimated from XRF intensities in the range 0–44 keV.
3. MIR-XRF_HYB1_	A sequential approach. PLSR using a concatenated matrix of the MIR-PLSR prediction (from variant 1) and XRF elemental contents.
4. MIR-XRF_HYB2_	A sequential approach. MIR-PLSR (variant 1) followed by PLSR prediction of the residuals using XRF elemental contents, and summation of the MIR estimate of the property and XRF estimate of the residual.
5. MIR-XRF_AV_	A high-level approach. Arithmetic averaging of predictions from variants 1 and 2.
6. MIR-XRF_GR_	A high-level approach. Granger-Ramanathan averaging of predictions from variants 1 and 2.

**Table 2:** Average performance in cross-validation (CV) and validation (V) of model variants for prediction of total organic carbon (OC), N, texture, pH, labile OC, and cation exchange capacity (CEC). See Table 1 for variant definitions. The best single and fusion approaches in training and testing for each property are shaded. RMSE = root mean squared error, RPIQ = ratio of prediction to interquartile distance, RI = relative improvement in validation.

Property	Variant	RMSECV		RPIQCV		RMSEV		RPIQV		RI (%)
		**Avg**	St dev	**Avg**	St dev	**Avg**	St dev	**Avg**	St dev	
**total OC**	MIR	**0.20**	0.01	**11.72**	2.64	**0.26**	0.06	**7.72**	1.76	
(g kg^−1^)	XRF	**0.68**	0.13	**3.44**	0.84	**0.78**	0.02	**2.66**	1.34	
	MIR-XRF_HYB1_	**0.26**	0.02	**8.94**	2.15	**0.31**	0.04	**6.59**	2.32	
	MIR-XRF_HYB2_	**0.21**	0.01	**10.86**	2.33	**0.26**	0.08	**7.62**	1.37	
	MIR-XRF_AV_	**0.37**	0.06	**6.20**	1.37	**0.43**	0.02	**4.83**	2.41	
	MIR-XRF_GR_	**0.20**	0.01	**11.78**	2.58	**0.26**	0.06	**7.82**	1.90	1.3
**total N**	MIR	**0.027**	0.002	**7.40**	1.69	**0.037**	0.007	**5.04**	1.48	
(g kg^−1^)	XRF	**0.061**	0.008	**3.27**	0.94	**0.070**	0.007	**2.71**	1.02	
	MIR-XRF_HYB1_	**0.060**	0.008	**3.29**	0.96	**0.069**	0.007	**2.73**	1.02	
	MIR-XRF_HYB2_	**0.028**	0.003	**6.97**	1.44	**0.039**	0.007	**4.72**	1.06	
	MIR-XRF_AV_	**0.036**	0.005	**5.40**	1.23	**0.045**	0.007	**4.17**	1.45	
	MIR-XRF_GR_	**0.026**	0.003	**7.53**	1.52	**0.037**	0.007	**4.99**	1.25	-1.0
**clay** (%)	MIR	**0.77**	0.03	**3.85**	0.72	**0.86**	0.02	**2.82**	0.47	
	XRF	**0.70**	0.04	**4.25**	0.99	**0.75**	0.12	**3.37**	1.15	
	MIR-XRF_HYB1_	**0.70**	0.02	**4.26**	0.87	**0.73**	0.11	**3.47**	1.12	3.1
	MIR-XRF_HYB2_	**0.79**	0.02	**3.76**	0.65	**0.87**	0.05	**2.84**	0.63	
	MIR-XRF_AV_	**0.68**	0.02	**4.36**	0.93	**0.74**	0.08	**3.38**	0.94	
	MIR-XRF_GR_	**0.67**	0.03	**4.42**	0.97	**0.73**	0.09	**3.41**	1.01	
**silt** (%)	MIR	**0.88**	0.04	**3.13**	0.49	**0.96**	0.19	**2.18**	0.66	
	XRF	**0.71**	0.04	**3.90**	0.86	**0.69**	0.11	**2.97**	0.71	
	MIR-XRF_HYB1_	**0.73**	0.05	**3.79**	0.82	**0.71**	0.12	**2.93**	0.87	
	MIR-XRF_HYB2_	**0.85**	0.08	**3.26**	0.80	**0.84**	0.09	**2.41**	0.45	
	MIR-XRF_AV_	**0.73**	0.04	**3.75**	0.73	**0.76**	0.15	**2.76**	0.85	
	MIR-XRF_GR_	**0.70**	0.04	**3.94**	0.85	**0.69**	0.12	**2.98**	0.80	0.3
**sand** (%)	MIR	**0.61**	0.03	**1.84**	0.60	**0.76**	0.04	**1.53**	0.71	
	XRF	**0.58**	0.06	**1.91**	0.61	**0.69**	0.13	**1.75**	1.03	
	MIR-XRF_HYB1_	**0.57**	0.05	**1.94**	0.6	**0.68**	0.14	**1.82**	1.11	
	MIR-XRF_HYB2_	**0.61**	0.03	**1.81**	0.57	**0.73**	0.13	**1.58**	0.63	
	MIR-XRF_AV_	**0.56**	0.05	**1.99**	0.61	**0.67**	0.10	**1.83**	1.12	4.6
	MIR-XRF_GR_	**0.55**	0.05	**2.00**	0.60	**0.68**	0.10	**1.80**	1.07	
**pH**	MIR	**0.12**	0.01	**4.16**	0.69	**0.16**	0.03	**3.45**	1.46	
	XRF	**0.18**	0.02	**2.88**	0.41	**0.21**	0.04	**2.49**	0.67	
	MIR-XRF_HYB1_	**0.15**	0.02	**3.62**	0.83	**0.18**	0.03	**2.94**	0.98	
	MIR-XRF_HYB2_	**0.13**	0.01	**3.94**	0.81	**0.15**	0.02	**3.55**	1.44	
	MIR-XRF_AV_	**0.13**	0.02	**4.01**	0.60	**0.15**	0.02	**3.56**	0.82	3.2
	MIR-XRF_GR_	**0.12**	0.01	**4.37**	0.66	**0.15**	0.01	**3.44**	1.13	
**labile OC**	MIR	**0.31**	0.01	**2.33**	0.11	**0.38**	0.03	**1.83**	0.19	
(g kg^−1^)	XRF	**0.37**	0.03	**1.92**	0.10	**0.39**	0.03	**1.76**	0.08	
	MIR-XRF_HYB1_	**0.34**	0.02	**2.10**	0.06	**0.35**	0.04	**2.00**	0.28	9.3
	MIR-XRF_HYB2_	**0.33**	0.02	**2.15**	0.09	**0.38**	0.05	**1.84**	0.32	
	MIR-XRF_AV_	**0.31**	0.02	**2.31**	0.09	**0.36**	0.01	**1.93**	0.06	
	MIR-XRF_GR_	**0.30**	0.02	**2.39**	0.11	**0.37**	0.02	**1.90**	0.14	
**CEC**	MIR	**11.3**	2.3	**1.80**	0.70	**13.6**	2.5	**1.51**	0.73	
(cmol_c_ kg^−1^)	XRF	**10.4**	0.5	**1.88**	0.41	**12.1**	1.4	**1.65**	0.72	
	MIR-XRF_HYB1_	**9.8**	1.9	**2.07**	0.77	**12.9**	0.8	**1.56**	0.74	
	MIR-XRF_HYB2_	**10.9**	2.0	**1.87**	0.69	**11.8**	3.7	**1.85**	1.02	12.1
	MIR-XRF_AV_	**9.6**	1.6	**2.09**	0.73	**11.2**	1.6	**1.78**	0.73	
	MIR-XRF_GR_	**9.4**	1.5	**2.14**	0.74	**11.4**	1.6	**1.76**	0.76	

## Data Availability

The data presented in this study are available on request from the corresponding author.

## References

[1] Drobnik T., Greiner L., Keller A., Grêt-Regamey A. (2018). Soil quality indicators—From soil functions to ecosystem services. Ecol. Indic..

[2] Adamchuk V.I., Ferguson R.B., Hergert G.W., Oerke E.-C., Gerhards R., Menz G., Sikora R.A. (2010). Soil Heterogeneity and Crop Growth. Precision Crop Protection—The Challenge and Use of Heterogeneity.

[3] Kuang B., Mahmood H.S., Quraishi M.Z., Hoogmoed W.B., Mouazen A.M., van Henten E.J. (2012). Sensing Soil Properties in the Laboratory, In Situ, and On-Line. Adv. Agron..

[4] Greenberg I., Linsler D., Vohland M., Ludwig B. (2020). Robustness of visible near-infrared and mid-infrared spectroscopic models to changes in the quantity and quality of crop residues in soil. Soil Sci. Soc. Am. J..

[5] Viscarra Rossel R.A., Walvoort D., McBratney A.B., Janik L.J., Skjemstad J.O. (2006). Visible, near infrared, mid infrared or combined diffuse reflectance spectroscopy for simultaneous assessment of various soil properties. Geoderma.

[6] Ellerbrock R.H., Gerke H.H. (2013). Characterization of Organic Matter Composition of Soil and Flow Path Surfaces Based on Physicochemical Principles—A Review. Adv. Agron..

[7] Soriano-Disla J.M., Janik L.J., Viscarra Rossel R.A., Macdonald L.M., McLaughlin M.J. (2014). The Performance of Visible, Near-, and Mid-Infrared Reflectance Spectroscopy for Prediction of Soil Physical, Chemical, and Biological Properties. Appl. Spectrosc. Rev..

[8] Stenberg B., Viscarra Rossel R.A., Mouazen A.M., Wetterlind J. (2010). Visible and Near Infrared Spectroscopy in Soil Science. Adv. Agron..

[9] Munnaf M.A., Nawar S., Mouazen A.M. (2019). Estimation of Secondary Soil Properties by Fusion of Laboratory and On-Line Measured Vis–NIR Spectra. Remote Sens..

[10] Marín-González O., Kuang B., Quraishi M.Z., Munóz-García M.Á., Mouazen A.M. (2013). On-line measurement of soil properties without direct spectral response in near infrared spectral range. Soil Tillage Res..

[11] Nocita M., Stevens A., van Wesemael B., Aitkenhead M., Bachmann M., Barthès B., Ben Dor E., Brown D.J., Clairotte M., Csorba A. (2015). Soil Spectroscopy: An Alternative to Wet Chemistry for Soil Monitoring. Adv. Agron..

[12] Greenberg I., Seidel M., Vohland M., Ludwig B. (2022). Performance of field-scale lab vs in situ visible/near- and mid-infrared spectroscopy for estimation of soil properties. Eur. J. Soil Sci..

[13] Haschke M., Flock J., Haller M. (2021). X-ray Fluorescence Spectroscopy for Laboratory Applications.

[14] Brouwer P. (2010). Theory of XRF..

[15] Weindorf D.C., Paulette L., Man T. (2013). In-situ assessment of metal contamination via portable X-ray fluorescence spectroscopy: Zlatna, Romania. Environ. Pollut..

[16] Nawar S., Cipullo S., Douglas R.K., Coulon F., Mouazen A.M. (2020). The applicability of spectroscopy methods for estimating potentially toxic elements in soils: State-of-the-art and future trends. Appl. Spectrosc. Rev..

[17] Wang S., Li W., Li J., Liu X. (2013). Prediction of Soil Texture Using FT-NIR Spectroscopy and PXRF Spectrometry With Data Fusion. Soil Sci..

[18] O’Rourke S.M., Stockmann U., Holden N.M., McBratney A.B., Minasny B. (2016). An assessment of model averaging to improve predictive power of portable vis-NIR and XRF for the determination of agronomic soil properties. Geoderma.

[19] Xu D., Zhao R., Li S., Chen S., Jiang Q., Zhou L., Shi Z. (2019). Multi-sensor fusion for the determination of several soil properties in the Yangtze River Delta, China. Eur. J. Soil Sci..

[20] O’Rourke S.M., Minasny B., Holden N.M., McBratney A.B. (2016). Synergistic Use of Vis-NIR, MIR, and XRF Spectroscopy for the Determination of Soil Geochemistry. Soil Sci. Soc. Am. J..

[21] Morona F., Dos Santos F.R., Brinatti A.M., Melquiades F.L. (2017). Quick analysis of organic matter in soil by energy-dispersive X-ray fluorescence and multivariate analysis. Appl. Radiat. Isot..

[22] Towett E.K., Shepherd K.D., Sila A., Aynekulu E., Cadisch G. (2015). Mid-Infrared and Total X-Ray Fluorescence Spectroscopy Complementarity for Assessment of Soil Properties. Soil Sci. Soc. Am. J..

[23] Tavares T.R., Molin J.P., Nunes L.C., Alves E.E.N., Melquiades F.L., de Carvalho H.W.P., Mouazen A.M. (2020). Effect of X-Ray Tube Configuration on Measurement of Key Soil Fertility Attributes with XRF. Remote Sens..

[24] Sharma A., Weindorf D.C., Man T., Aldabaa A.A.A., Chakraborty S. (2014). Characterizing soils via portable X-ray fluorescence spectrometer: 3. Soil reaction (pH). Geoderma.

[25] Weindorf D.C., Chakraborty S., Herrero J., Li B., Castañeda C., Choudhury A. (2016). Simultaneous assessment of key properties of arid soil by combined PXRF and Vis-NIR data. Eur. J. Soil Sci..

[26] Wang D., Chakraborty S., Weindorf D.C., Li B., Sharma A., Paul S., Ali M.N. (2015). Synthesized use of VisNIR DRS and PXRF for soil characterization: Total carbon and total nitrogen. Geoderma.

[27] Tavares T.R., Molin J.P., Javadi S.H., de Carvalho H.W.P., Mouazen A.M. (2020). Combined Use of Vis-NIR and XRF Sensors for Tropical Soil Fertility Analysis: Assessing Different Data Fusion Approaches. Sensors.

[28] Bates J.M., Granger C.W.J. (1969). The Combination of Forecasts. Oper. Res. Soc..

[29] Granger C.W.J., Ramanathan R. (1984). Improved methods of combining forecasts. J. Forecast..

[30] Crawley M.J. (2015). Statistics: An Introduction Using R.

[31] IUSS Working Group WRB (2015). World Reference Base for Soil Resources 2014, Update 2015: International Soil Classification System for Naming Soils and Creating Legends for Soil Maps.

[32] Koch H.-J., Dieckmann J., Büchse A., Märländer B. (2009). Yield decrease in sugar beet caused by reduced tillage and direct drilling. Eur. J. Agron..

[33] (2002). Soil Quality—Determination of Particle Size Distribution in Mineral Soil Material—Method by Sieving and Sedimentation (ISO 11277:1998 + ISO 11277:1998 Corrigendum 1:2002).

[34] (2005). Soil Quality—Determination of pH (ISO 10390:2005).

[35] Koenig N., Fortmann H. (1996). Probenvorbereitungs-, Untersuchungs-und Elementbestimmungs-Methoden des Umweltanalytik-Labors der Niedersaechsischen Forstlichen Versuchsanstalt und des Zentrallabor 2 des Forschungszentrums Waldoekosysteme.

[36] Zimmermann M., Leifeld J., Fuhrer J. (2007). Quantifying soil organic carbon fractions by infrared-spectroscopy. Soil Biol. Biochem..

[37] Von Lützow M., Kögel-Knabner I., Ekschmitt K., Flessa H., Guggenberger G., Matzner E., Marschner B. (2007). SOM fractionation methods: Relevance to functional pools and to stabilization mechanisms. Soil Biol. Biochem..

[38] Poeplau C., Don A., Six J., Kaiser M., Benbi D., Chenu C., Cotrufo M.F., Derrien D., Gioacchini P., Grand S. (2018). Isolating organic carbon fractions with varying turnover rates in temperate agricultural soils—A comprehensive method comparison. Soil Biol. Biochem..

[39] Beckhoff B., Kanngießer B., Langhoff N., Wedell R., Wolff H. (2006). Handbook of Practical X-ray Fluorescence Analysis.

[40] Zhu Y., Weindorf D.C., Zhang W. (2011). Characterizing soils using a portable X-ray fluorescence spectrometer: 1. Soil texture. Geoderma.

[41] Sharma A., Weindorf D.C., Wang D., Chakraborty S. (2015). Characterizing soils via portable X-ray fluorescence spectrometer: 4. Cation exchange capacity (CEC). Geoderma.

[42] Pospiech S. (2018). Geochemical Characterization of Tea Leaves (*Camellia sinensis*) and Soils for Provenance Studies Based on Compositional Data Analysis. Ph.D. Dissertation.

[43] Ruppert H. (1991). Natürliche Spurenmetallgehalte im Boden und ihre anthropogene Überprägung. Mittelungen Osterr. Geol. Ges..

[44] Dean J.R. (2014). Environmental Trace Analysis: Techniques and Applications.

[45] Govindaraju K. (1994). Compilation of working values and sample description for 383 Geostandards. Geostand. Geoanalytical Res..

[46] Mevik B.-H., Wehrens R., Liland K.H. pls: Partial least squares and principal component regression. https://CRAN.R-project.org/package=pls.

[47] Wehrens R. (2020). Chemometrics with R: Multivariate Data Analysis in the Natural and Life Sciences.

[48] Stevens A., Ramirez-Lopez L. An Introduction to the Prospectr Package. https://cran.r-project.org/web/packages/prospectr/vignettes/prospectr.html.

[49] Viscarra Rossel R.A., Behrens T. (2010). Using data mining to model and interpret soil diffuse reflectance spectra. Geoderma.

[50] Chang C.-W., Laird D.A., Mausbach M.J., Hurburgh C.R. (2001). Near-infrared reflectance spectroscopy–Principal components regression analyses of soil properties. Soil Sci. Soc. Am. J..

[51] Chakraborty S., Weindorf D.C., Li B., Ali Aldabaa A.A., Ghosh R.K., Paul S., Nasim Ali M. (2015). Development of a hybrid proximal sensing method for rapid identification of petroleum contaminated soils. Sci. Total Environ..

[52] Weiss C.E., Roetzet G.R. GeomComb: (Geometric) Forecast Combination Methods. https://cran.r-project.org/web/packages/GeomComb/index.html.

[53] Mehmood T., Liland K.H., Snipen L., Sæbø S. (2012). A review of variable selection methods in Partial Least Squares Regression. Chemom. Intell. Lab. Syst..

[54] Liland K.H., Mehmood T., Sæbø S. (2020). plsVarSel: Variable Selection in Partial Least Squares. https://CRAN.R-project.org/package=plsVarSel.

[55] Chong I.-G., Jun C.-H. (2005). Performance of some variable selection methods when multicollinearity is present. Chemom. Intell. Lab. Syst..

[56] Kelley K.R., Stevenson F.J. (1995). Forms and nature of organic N in soil. Fertil. Res..

[57] Ludwig B., Wölfel P., Greenberg I., Piepho H.-P., Spörlein P. (2022). Application of mixed-effects modelling and rule-based models to explain copper variation in soil profiles of southern Germany. Eur. J. Soil Sci..

[58] Brady N.C., Weil R.R. (2016). The Nature and Properties of Soils.

[59] Agbenin J.O., Olojo L.A. (2004). Competitive adsorption of copper and zinc by a Bt horizon of a savanna Alfisol as affected by pH and selective removal of hydrous oxides and organic matter. Geoderma.

[60] Collin S., Baskar A., Geevarghese D.M., Ali M.N.V.S., Bahubali P., Choudhary R., Lvov V., Tovar G.I., Senatov F., Koppala S. (2022). Bioaccumulation of lead (Pb) and its effects in plants: A review. J. Hazard. Mater. Lett..

[61] Silva S.H.G., Teixeira A.F.d.S., de Menezes M.D., Guilherme L.R.G., Moreira F.M.d.S., Curi N. (2017). Multiple linear regression and random forest to predict and map soil properties using data from portable X-ray fluorescence spectrometer (pXRF). Ciênc. Agrotec..

[62] Sparks D.L. (2003). Environmental Soil Chemistry.

[63] Essington M.E. (2004). Soil and Water Chemistry: An Integrative Approach.

[64] Ludwig B., Sawallisch A., Heinze S., Joergensen R.G., Vohland M. (2015). Usefulness of middle infrared spectroscopy for an estimation of chemical and biological soil properties—Underlying principles and comparison of different software packages. Soil Biol. Biochem..

[65] Javadi S.H., Munnaf M.A., Mouazen A.M. (2021). Fusion of Vis-NIR and XRF spectra for estimation of key soil attributes. Geoderma.

